# Psychometric properties and factor structure of the Children's Eating Behavior Questionnaire in a Danish sample of children with overweight and obesity

**DOI:** 10.1007/s40519-025-01798-1

**Published:** 2025-11-21

**Authors:** Dorthe Dalstrup Pauls, Caroline Bruun Abild, Loa Clausen, Jens Meldgaard Bruun

**Affiliations:** 1 Steno Diabetes Center Aarhus, Aarhus University Hospital, Palle Juul-Jensens Boulevard 11, Entrance A, 8200 Aarhus N, Denmark; 2 Department of Clinical Medicine, Aarhus University, 8200 Aarhus N, Denmark; 3 Danish National Center for Obesity, 8200 Aarhus N, Denmark; 4 Department of Child and Adolescent Psychiatry, Aarhus University Hospital, 8200 Aarhus N, Denmark

**Keywords:** Eating Behavior Traits, Children's Eating Behavior Questionnaire, Validation, Obesity, Adiposity, Children and Adolescents

## Abstract

**Purpose:**

The Children's Eating Behavior Questionnaire (CEBQ) is a reliable and widely used tool to assess eating behavior traits in children. However, currently no Danish version of the CEBQ exists. This study aimed to translate the CEBQ into Danish and investigate its psychometric properties and factor structure in children with overweight and obesity. Secondly, differences in eating behavior traits between children with overweight and obesity were explored.

**Methods:**

Children (7–14 years) were recruited from a 10-week multicomponent lifestyle camp. Parents completed the CEBQ with their child before camp, and anthropometry was measured. CEBQ is scored from 1 to 5, with higher scores indicating a higher tendency toward a specific behavior. A confirmatory factor analysis (CFA) was performed to test the original eight-factor structure. Internal reliability was assessed using McDonald’s Omega.

**Results:**

In total, 190 children (12.3 ± 1.36 years) participated. The CFA confirmed the eight-factor model, with all items loading significantly on their respective factors. Internal reliability was acceptable for the full CEBQ scale (ω = 0.85) and most subscales (ω ≥ 0.70) but only moderate for Satiety Responsiveness (ω = 0.59) and Emotional Undereating (ω = 0.65). No statistically significant difference was found between children with overweight and obesity according to the Bonferroni-adjusted alpha.

**Conclusions:**

The Danish CEBQ is a valid tool to assess eating behavior traits in Danish children with overweight and obesity; although, generalizability may be limited due to a relatively small sample size. Future studies should further validate the Danish CEBQ by assessing test–retest reliability, construct validity, and factor structure in a generalizable sample across weight categories.

**Level of evidence:**

Level V, Cross-sectional, Psychometric study.

## Introduction

Globally, according to projected numbers, 28% of children are living with overweight and obesity in 2025, and by 2035 these numbers are estimated to reach 39% [1]. It is well-established that the majority of children with obesity will continuously have obesity in adolescence and adulthood [2, 3], which altogether increases the global risk of comorbidities such as type 2 diabetes, cardiovascular diseases, and premature mortality [4, 5]. For decades, body mass index (BMI = kg/m^2^) adjusted for sex and age has been widely used to identify children at risk of developing obesity and monitor the effect of childhood obesity treatment. The continued global increase in obesity may, however, suggest a need for deeper insights into behavioral characteristics associated with weight development.

Eating behavior is established early in life through a complex interplay of genetic, social, cultural, environmental, and economic factors [6–9], and several studies have demonstrated an association between eating behavior characteristics and childhood obesity [10–12]. In theory, children are born with the ability to self-regulate their eating based on internal cues of hunger, satiation, and satiety, arising from and maintained by a series of overlapping mediating physiological processes (e.g. hormones, neurotransmitters, and polypeptides) referred to as ‘the satiety cascade’ [13]. However, throughout childhood, these implicit processes are modulated by non-homeostatic factors involved in appetite control, such as food hedonics (a reward driven appetite/pleasure of food) [14]. Numerous theoretical models have been developed to explore a person’s eating style or disposition to eat, typically referred to as eating behavior traits [15]. A prospective study by Warkentin et al. have demonstrated that fat mass and waist-to-height ratio at age 7 predict greater food approach at age 10 (Food Responsiveness, Enjoyment of Food, Desire to Drink and Emotional Overeating), whereas a reversed association was shown for waist-to weight ratio, suggesting that greater appetite avidity at age 7 predict higher central adiposity three years later [16]. In children and adolescents participating in a 10-week lifestyle intervention, a study showed that those with the highest food approach behavior before the intervention maintained a higher BMI-SDS one year later, emphasizing the need for long-term support in this subgroup of children with overweight and obesity to prevent continued weight gain into adulthood [17]. Thus, a deeper insight into eating behavior traits in children and adolescents may provide valuable knowledge to identify those at risk of developing overweight and obesity, and tailor future intervention strategies.

Currently, a validated instrument for assessing eating behavior traits in Danish children does not yet exist. Therefore, the primary aim of the present study is to translate the Children's Eating Behavior Questionnaire (CEBQ) into Danish and test its psychometric properties and factor structure, in order to enable future assessment of eating behaviors traits in Danish children. Secondly, the study will explore differences in eating behavior traits between children with overweight and obesity.

## Materials and methods

The present study is based on baseline data from The COPE study, which is a prospective, nonrandomized intervention study designed to investigate the effect of a higher protein diet during lifestyle camp on weight development in 7–14-year-olds with overweight and obesity. The complete study design, the effect of a higher protein diet during camp, and overall short and long-term changes in body composition, quality of life and overeating/loss-of-control eating following the intervention, has been described in two previous papers [18, 19]. Briefly, all children assigned to the camp from October 2020 to March 2022 were invited to participate. The camp has a multicomponent approach, and the primary aim of the camp is to improve health and quality of life in children. Children were referred by their general practitioner to attend the camp. Children were excluded from the study if they had a diagnosis or an eating disorder requiring a special diet or if they were categorized as having normal weight (BMI-SDS ≤ 1SD) at baseline. Children or parents/guardians who participated in another clinical trial or did not understand or were unwilling or unable to comply with the study protocol were also excluded.

### The Children's Eating Behavior Questionnaire (CEBQ)

The CEBQ is a comprehensive widely used parent-reported questionnaire, which has shown good reliability and continuity in assessing eating behavior throughout childhood [20, 21]. The CEBQ was originally developed in UK by Wardle et al. to assess normal variation in eating behaviors among 2–9-year-old children, but it has currently been validated in children from 1–13 years of age, primarily in a mixed population of children with normal weight, overweight, and obesity [20]. In the present sample of 7–14-year-olds, many meals are still consumed within the family, thus parental reports remain relevant. Furthermore, a self-reported version, namely the Adult Eating Behavior Questionnaire (AEBQ), is only available to assess eating behavior traits in adolescents ≥ 13 years of age, supporting the validation of CEBQ in the current sample [22]. To our knowledge, eight different countries including Holland [12], China [23], and Spain [11] have translated and validated the CEBQ, with most studies showing good internal consistency, test–retest reliability, and construct validity [11, 12, 20, 23–29].

The CEBQ consist of 35 items divided into eight eating behavior traits; Food Responsiveness (FR), Emotional Overeating (EOE), Enjoyment of Food (EF), Desire to Drink (DD), Satiety Responsiveness (SR), Slowness in Eating (SE), Emotional Undereating (EUE) and Food Fussiness (FF). Parents are asked to rate their child’s eating behavior on a five-point Likert scale (never, rarely, sometimes, often, or always). Responses were scored and categorized according to tool specific guidelines set by the original author i.e., each item was scored from 1 (never) to 5 (always), with items 3, 4, 10, 16 and 32 reverse scored. For each eating behavior trait scale, the mean score of items was calculated, resulting in a score ranging from 1 to 5. Higher scores indicate a higher tendency of the specific behavior. Additionally, the scales can be categorized as food-approaching behaviors (FR, EOE, EF, DD) and food-avoidant behaviors (SR, SE, EUE, FF) [20].

In the present study, the CEBQ was translated into Danish by using a professional translation company (www.vidkom.dk). Two translators with experience in health communication performed the translation. The initial translation from English to Danish was done by a native Danish speaker, while the back-translation was carried out by a native English speaker. The translation process and possible discrepancies between translators were discussed at a meeting between the two translators. A consensus report was developed explaining discrepancies and displaying comments by the translators. Based on the consensus report, the authors decided on the final Danish version of CEBQ.

In the present study, the CEBQ was delivered electronically to the participating parents/guardians using www.project-redcap.org database located at Aarhus University. Participating parents/guardians were encouraged to answer the CEBQ with their child before starting the lifestyle camp.

A license to translate and use the CEBQ was granted by the original author, (University College London https://xip.uclb.com) [20].

### Anthropometry

Body weight (kg), body fat (%), and skeletal muscle mass (kg) were measured using a Bioelectric impedance (InBody model 270, InBody Denmark). Height (m) was measured using a fixed wall measuring tape. BMI-SDS was calculated using World Health Organization AnthroPlus software. Children with a BMI-SDS > 1SD were categorized as having overweight, and children with a BMI-SDS > 2SD were categorized as having obesity [30].

Camp staff measured all children within the first week of camp.

### Statistics

A CFA using a diagonally weighted least squares (DWLS) estimator was performed to test the original eight-factor structure, as the data were ordinal and non-normally distributed. As recommended by Hu and Bentler [31], model fit was assessed using root mean squared error of approximation (RMSEA), comparative fit index (CFI), Tucker-Lewis index (TLI), and standardized root mean squared residual (SRMR). At least two of the following cut-offs should be fulfilled to consider a good model fit to perform a confirmatory factor analysis (CFA): RMSEA ≤ 0.06, CFI > 0.96, TLI > 0.96 and SRMR ≤ 0.10 [31].

Internal reliability was estimated for the full CEBQ scale and each subscale using a McDonald’s Omega test. A McDonald’s Omega ≥ 0.70 was considered satisfactory.

Correlations between the eight factors (subscales) and between the eight factors and BMI-SDS were preferably assessed using a Pearson’s correlation test. QQ-plots were used to assess normality, and the linearity of relationships between variables was evaluated using scatterplots and simple linear regression analyses. In cases where the assumptions of normality or linearity were violated, Spearman’s rank correlation was applied instead. Correlation coefficients from 0 to ≤ 0.3 were considered weak, ≥ 0.3 to ≤ 0.5 were considered moderate, and ≥ 0.5 were considered strong [32].

Simple linear regression analyses with Welch’s correction were performed to investigate differences in each eating behavior trait between children with overweight and obesity at baseline. QQ-plots of the residuals were used to check for normal distribution errors. Effect sizes were calculated as Hedges’ g with 99% confidence intervals (CIs).

Continuous data are presented for the complete sample as mean ± standard deviation (SD) and for the groups (overweight vs. obesity) as mean ± standard error (SE). Categorical data are displayed as absolute numbers and percentages [n (%)]. To account for multiple testing in the correlation analyses, a Bonferroni-corrected significance threshold of p < 0.006 (0.05/8 subscales) was considered statistically significant. The Bonferroni alpha correction was also applied when testing differences between children with overweight and obesity.

CFA was performed using lavaan 0.6.14 [33]. CFA and goodness of fit statistics were performed using R version 4.1.1 (RStudio, Inc. USA). All other statistics were performed using Stata/MP 17.0 (StataCorp LLC, USA).

## Results

In total, 190 participants answered the CEBQ before camp. Children included had a mean age of 12.3 ± 1.36 years, 57.9% were girls, and 83% of the children had obesity entering camp (Table 1).

**Table 1:** Baseline characteristics

	All (n:190) Mean ± SD
Age	12.3 ± 1.36
Sex (M/F)	80/110
**Eating behavior**	
Food Responsiveness (FR)	3.5 ± 0.94
Emotional Overeating (EOE)	2.8 ± 0.95
Enjoyment of Food (EF)	4.1 ± 0.62
Desire to Drink (DD)	2.8 ± 0.95
Satiety Responsiveness (SR)	2.2 ± 0.62
Slowness in Eating (SE)	2.3 ± 0.85
Emotional Undereating (EUE)	2.4 ± 0.74
Food Fussiness (FF)	2.6 ± 0.98
**Anthropometry (n:187)**	
Weight (kg)	73.1 ± 15.77
Height (cm)	1.59 ± 0.09
BMI-SDS (WHO)	2.6 ± 0.68
*Overweight* > *1SD (17%)*	1.6 ± 0.30
*Obesity* > *2SD (83%)*	2.8 ± 0.56
Body fat %	41.3 ± 6.61 (n:178)
Skeletal muscle mass (kg)	23.1 ± 5.01 (n:178)

### Factor analysis and internal reliability of the Danish CEBQ

The goodness of fit statistics (RMSEA 0.10 [90% confidence interval (CI) 0.091;0.102]; CFI 0.96; TLI 0.95; SRMR 0.10) was acceptable, revealing an adequate fit of the original CEBQ subscales for the present sample.

Based on the CFA, the eight factors explained 84.3% of the common variance. All items loaded significantly on their respective factors, with most factor loadings ≥ 0.70, some loadings between ≥ 0.50–0.60, and three loadings < 0.40. Item 30 (*My child cannot eat a meal if s/he has had a snack just before)* loaded < 0.30 on the Satiety Responsiveness scale. Excluding item 30, only led to a marginal increase in internal reliability (from ω = 0.59 to ω = 0.63). Therefore, we chose to retain the item to preserve the conceptual integrity of the scale. The Satiety Responsiveness scale had a moderate internal reliability, probably because of low loadings for item 30 *(My child cannot eat a meal if s/he has had a snack just before)* and item 17 *(My child leaves food on his/her plate at the end of a meal)* (Table 2).

**Table 2:** Loading factors for CEBQ items estimated with a confirmatory factor analysis (n:190)

Factor	Variance %	Scale and items	Factor loadings	McDonald’s Omega
Factor 1	15.2%	Food Responsiveness (FR)		0.88
		12. My child is always asking for food	0.82	
		14. If allowed to, my child would eat too much	0.85	
		19. Given the choice, my child would eat most of the time	0.90	
		28. Even if my child is full up s(he) find room to eat his/her favorite food	0.69	
		34. If given the chance, my child would always have food in his/her mouth	0.88	
Factor 2	10.2%	Emotional Overeating (EOE)		0.82
		2. My child eats more when worried	0.80	
		13. My child eats more when annoyed	0.76	
		15. My child eats more when anxious	0.70	
		27. My child eats more when s/he has nothing else to do	0.91	
Factor 3	8.1%	Enjoyment of Food (EF)		0.77
		1.My child loves food	0.73	
		5. My child is interested in food	0.71	
		20. My child looks forward to mealtimes	0.86	
		22. My child enjoys eating	0.66	
Factor 4	4.5%	Desire to Drink (DD)		0.78
		6. My child is always asking for a drink	0.62	
		29. If given the chance, my child would drink continuously throughout the day	0.85	
		31. If given the chance, my child would always be having a drink	0.88	
Factor 5	14.5%	Satiety Responsiveness (SR)		0.59
		3. My child has a big appetite	0.98	
		17. My child leaves food on his/her plate at the end of a meal	0.34	
		21. My child gets full before his/her meal is finished	0.57	
		26. My child gets full up easily	0.67	
		30. My child cannot eat a meal if s/he has had a snack just before	0.25	
Factor 6	12.6%	Slowness in Eating (SE)		0.77
		4. My child finishes his/her meal quickly	0.95	
		8. My child eats slowly	0.84	
		18. My child takes more than 30 min. to finish a meal	0.66	
		35. My child eats more and more slowly during the course of a meal	0.39	
Factor 7	5.0%	Emotional Undereating (EUE)		0.65
		9. My child eats less when angry	0.57	
		11. My child eats less when s/he is tired	0.51	
		23. My child eats more when h/she is happy	0.79	
		25. My child eats less when upset	0.55	
Factor 8	14.2%	Food Fussiness (FF)		0.90
		7. My child refuses new foods at first	0.83	
		10. My child enjoys tasting new foods	0.89	
		16. My child enjoys a wide variety of foods	0.78	
		24. My child is difficult to please with meals	0.64	
		32. My child is interested in tasting food s/he has not tasted before	0.92	
		33. My child decides that s/he does not like a food, even without tasting it	0.77	

### Correlation between scales

When evaluating the subscales, the food approach scales, as expected, tended to be positively (moderate to strong) inter-correlated and negatively (weak to moderate) correlated with the food avoidant scales, except for Emotional Overeating and Emotional Undereating which was positively correlated. The strongest correlation was found between Food Responsiveness and Emotional Overeating (Pearson’s *r* = 0.62, p < 0.006) (Table 3). In general, BMI-SDS correlated positively with food approach scales and negatively with food avoidant scales, except for Food Fussiness. Significant positive correlations were found between BMI-SDS and Desire to Drink (Spearman’s *rho* = 0.24, p < 0.006), whereas Food Responsiveness (Spearman’s *rho* = 0.17, p < 0.05), Emotional Overeating (Spearman’s *rho* = 0.15, p < 0.05), and Food Fussiness (Spearman’s *rho* = 0.15, p < 0.05) tended to be positively correlated with BMI-SDS.

**Table 3:** Correlations between scores on different CEBQ scales (n:190)

	FR	EOE	EF	DD	SR	SE	EUE	FF
Food Responsiveness (FR)	1							
Emotional Overeating (EOE)	*r* = 0.62**	1						
Enjoyment of Food (EF)	*r* = 0.55**	*r* = 0.33**	1					
Desire to Drink (DD)	*r* = 0.39**	*r* = 0.28**	*r* = 0.22**	1				
Satiety Responsiveness (SR)	*r* = − 0.49**	*r* = − 0.17	*r* = − 0.44**	*rho* = − 0.03	1			
Slowness in Eating (SE)	*r* = − 0.29**	*rho* = − 0.09	*r* = − 0.24**	*rho* = − 0.07	*r* = 0.36**	1		
Emotional Undereating (EUE)	*rho* = 0.07	*r* = 0.32**	*rho* = − 0.03	*r* = 0.16	*r* = 0.23**	*rho* = 0.11	1	
Food Fussiness (FF)	*rho* = 0.07	*rho* = 0.07	*r* = -0.32**	*rho* = 0.02	*rho* = 0.03	*rho* = 0.03	*rho* = 0.11	1

*rho* = Spearman correlation coefficient

*r* = Pearson correlation coefficient

^**^p < *0.006*

### Eating behavior traits in children with overweight and obesity

No statistically significant differences were found between children with overweight and obesity according to the Bonferroni-adjusted alpha. Trends were observed, and some between-group differences reached significance when the alpha level was not adjusted. However, the effect sizes were only small to medium (Hedges’ g = 0.38–0.45) with large 99% CIs indicating that this study was not powered to assess significant differences in eating behavior traits between children with overweight and obesity (Table 4).

**Table 4:** Differences in eating behavior traits between children with overweight and obesity

	Overweight (n:31) Mean ± SE	Obesity (n:156) Mean ± SE	Differences between children with overweight (ref.) and obesity Δ [95% CI]	Hedges’ g g [99% CI]
Age	12.8 ± 0.19	12.2 ± 0.11	− 0.64 [− 1.08;− 0.21]	− 0.47 [− 0.98;0.03]
Sex (M/F)	10/21	68/88	p = 0.24	
**Eating behavior**	n:31	n:156	n:187	
Food Responsiveness (FR)	3.2 ± 0.18	3.6 ± 0.07	0.42 [0.04;0.79]	0.45 [− 0.06;0.96]
Emotional Overeating (EOE)	2.6 ± 0.17	2.8 ± 0.07	0.20 [− 0.17;0.56]	0.21 [− 0.29;0.72]
Enjoyment of Food (EF)	4.0 ± 0.11	4.1 ± 0.05	0.12 [− 0.11;0.36]	0.19 [− 0.31;0.70]
Desire to Drink (DD)	2.5 ± 0.16	2.9 ± 0.08	0.41 [0.07;0.75]	0.43 [− 0.08;0.94]
Satiety Responsiveness (SR)	2.4 ± 0.11	2.1 ± 0.05	− 0.21 [− 0.44;0.02]	− 0.34 [− 0.85;0.16]
Slowness in Eating (SE)	2.5 ± 0.15	2.3 ± 0.07	− 0.18 [− 0.50;0.14]	− 0.21 [− 0.72;0.29]
Emotional Undereating (EUE)	2.6 ± 0.16	2.4 ± 0.06	− 0.24 [− 0.57;0.09]	− 0.34 [− 0.84;0.17]
Food Fussiness (FF)	2.3 ± 0.16	2.7 ± 0.08	0.37 [0.02;0.72]	0.38 [− 0.13;0.89]

## Discussion

The present findings confirmed the original eight-factor model and showed adequate internal reliability, similar to the original manuscript [20], as well as other translations of the CEBQ [11, 12, 24]. However, the subscales Emotional Undereating and Satiety Responsiveness showed only moderate internal reliability, and two items loaded below 0.40 on the Satiety Responsiveness scale, similar to the Polish translation of the CEBQ [26]. Loadings below 0.50 may indicate reduced convergent validity, and the relevance of the items should therefore be considered [34]. Although, differences in factor loadings and the internal reliability between studies could also be caused by inaccurate translation or different populations being investigated. For example, in younger populations the scales may perform differently due to accelerated growth, and children with normal weight may tend to emotionally undereat rather than emotionally overeat in response to negative emotions. One previous study investigating school-age children with overweight and obesity suggested a three-factor structure of the CEBQ consisting of a reward-based eating scale (FR, EF, SR, and SE), an emotional eating scale (EOE and EUE), and a picky eating scale (FF), arguing that this three-factor model may be more clinically useful and easier to understand for children and parents seeking treatment for obesity [25]. In the present study, the correlations between factors partially support this three-factor structure of the CEBQ. However, this interpretation would overlook the strong positive correlation between Food Responsiveness and Emotional Overeating, as well as the moderate negative correlation between Food Fussiness and Enjoyment of Food, which may provide valuable insights for targeting childhood obesity treatment. Additionally, in the present sample, the eight-factor model showed satisfactory psychometric properties to evaluate eating behavior traits in Danish children with overweight and obesity. Therefore, an Exploratory Factor Analysis was considered unnecessary.

To further strengthen the validation of the Danish CEBQ, future studies should include additional validation methods, such as test–retest reliability and construct validity. Additionally, the psychometric properties of the Danish CEBQ should be evaluated in a Danish sample of children across multiple weight categories, including normal weight, and across multiple age-groups to strengthen its utility to monitor child development and identify risk profiles. In addition, assessing the psychometric properties in such samples could show whether the moderate internal reliability of the subscales Emotional Undereating and Satiety Responsiveness may be due to a relatively low frequency of "often" and "always" responses to items number 11 (*My child eats less when s/he is tired*), 17 (*My child leaves food on his/her plate at the end of a meal*), and 30 (*My child cannot eat a meal if s/he has had a snack just before*), which might be less frequent in samples with a higher body weight.

In this study, no differences were observed in eating behavior traits between children with overweight and obesity. Previous studies have shown that children with obesity have higher scores on Food Responsiveness compared to children with overweight [28, 35, 36]. Other studies, however, only reported Food Responsiveness to be higher in children with overweight/obesity compared to children with normal weight [11, 12] or find no linear association between Food Responsiveness and weight class [23, 27].

According to Wardle et al., higher scores on Desire to Drink reflect an increased desire to carry drinks along or drink continuously throughout the day [20]. One previous study investigating 7–9-year-old children found a positive linear trend between Desire to Drink and weight class [36]. However, in accordance with the present study, most studies do not replicate this finding [11, 24, 35]. One previous study found that Desire to Drink was only increased in children with overweight but not in children with obesity [35] and two studies did not report on associations between Desire to Drink and weight class [12, 29]. In total, three questions constitute the subscale Desire to Drink, e.g., item number 6 (*My child is always asking for a drink).* Wardle et al. included the Desire to Drinks subscale based on parental interviews, where some parents reported that their children wanted to carry drinks (usually sweetened drinks) with them. However, the three items assess general beverage-seeking behavior rather than the specific desire to drink sweetened drinks [20]. In future research, it may be relevant to explore how parents interpret the term ‘drink’ and whether responses primarily reflect a desire for sweetened beverages. Given current evidence linking higher intake of sugar-sweetened beverages to childhood obesity [37, 38] explicitly investigating the desire for sweetened drinks may be important for the treatment and prevention of childhood obesity.

Compared to children with normal weight, evidence suggests a correlation between overweight/obesity and lower Satiety Responsiveness [11, 12, 23, 28, 35], although this was not replicated in the present study. Previous studies have shown either no correlation [11, 12, 36] or a negative correlation between BMI-SDS and Food Fussiness, suggesting that picky eating is typically predictive of thinness and/or protective against becoming overweight [24, 39]. Overall, further research is needed to determine whether incorporating individual eating behavior traits into treatment approaches could enhance the effectiveness of childhood obesity interventions.

### Limitations

The present study has some limitations that should be acknowledged. First, the current Danish version of the CEBQ should be used with caution because translation always involve decisions regarding content, cultural, and grammatical considerations, which are important to take into account when adapting an instrument to a new culture. Second, additional validation methods e.g., test/retest reliability and construct validity would have provided a more detailed validation of the Danish CEBQ. In future validation studies of the Danish CEBQ, it would be valuable to include construct validity by assessing the correlation between the CEBQ and another validated questionnaire suitable to assess eating behavior traits in children e.g., The Dutch Eating Behavior Questionnaire or the Three-Factor Eating Behavior Questionnaire. Third, the CEBQ was originally developed for 2–9-year-old children, and is mostly validated in a mixed population of children with normal weight, overweight, and obesity. However, other studies have shown satisfactory psychometric properties in school-aged children across all weight classes [25–27, 29]. Fourth, the results on differences between children with overweight and obesity should be interpreted with caution due to the unbalanced group sizes. Finally, the relatively small sample size in this study increases the risk of random error and reduces the robustness of the validation process. Thus, larger validation studies with more generalizable samples are needed.

## Conclusion

Based on the present study, the Danish version of the CEBQ showed good psychometric properties to evaluate eating behavior traits in Danish children with overweight and obesity, adding to the general validity of the CEBQ. The confirmatory factor analysis supported the original eight-factor structure, and most factors showed adequate internal reliability. This first attempt to translate and assess the psychometric properties and factor structure of the Danish CEBQ is an important step towards understanding eating behavior traits in Danish children. Future research should include additional validation methods and investigate eating behavior traits across weight categories, with the aim of improving prevention and treatment strategies for childhood obesity.

## Data Availability

Deidentified individual participant data will be made available upon reasonable request. Further enquiries can be directed to the corresponding author.

## Abbreviations

CEBQ: Children's Eating Behavior Questionnaire
FR: Food responsiveness
EOE: Emotional overeating
EF: Enjoyment of food
DD: Desire to drink
SR: Satiety responsiveness
SE: Slowness in eating
EUE: Emotional undereating
FF: Food fussiness
